# Supplementary material for: Unravelling the Antifibrinolytic Mechanism of Action of the 1,2,3-Triazole Derivatives
Source: Int J Mol Sci. 2024 Jun 26;25(13):7002. doi: 10.3390/ijms25137002 (PMC11241262; doi:10.3390/ijms25137002)
Supplement: Supplementary file 1 [file ijms-25-07002-s001.zip › ijms-2980085-supplementary.pdf]

Table S1. Inhibition of plasmin's activity by tranexamic acid (TXA), compounds **1** and **5**, represented as the percentage of plasmin's inhibition determined at various concentrations of the tested compounds. Plasmin inhibition values were calculated based on the fluorescence slopes obtained from the plasmin activity assay. All datapoints are presented as mean ± SD. Compound **5** achieved its maximum solubility at 20 mM.

Concentration	Compound		
(mM)	TXA	1	5
0.5	2.61 ± 6.26	0.56 ± 4.92	-3.60 ± 6.18
5	12.32 ± 1.44	4.30 ± 3.02	6.29 ± 2.05
10	22.37 ± 1.38	7.36 ± 0.78	23.92 ± 2.74
15	28.00 ± 4.31	11.64 ± 6.34	31.31 ± 3.28
20	29.11 ± 1.96	48.00 ± 3.05	65.31 ± 10.56
30	53.06 ± 6.87	76.60 ± 14.25	-
50	59.24 ± 1.43	100.49 ± 0.23	-

Table S2. Effect of TXA and compounds **1** and **5** in tissue-type plasminogen activator (tPA) activity of hydrolyzing a synthetic substrate. Data is represented as the percentage of tPA's activity determined in the presence of different concentrations of the tested compounds. tPA activity values were extracted from the absorbance slopes obtained from the tPA activity assay. All datapoints are presented as mean ± SD.

Concentration	Compound		
(mM)	TXA	1	5
0.5	92.08 ± 3.54	96.53 ± 0.78	93.70 ± 1.14
1	89.90 ± 1.53	94.21 ± 4.17	91.18 ± 1.15
5	87.18 ± 0.98	96.95 ± 3.08	88.78 ± 2.89
10	87.33 ± 1.14	91.20 ± 2.43	89.39 ± 0.68
15	84.84 ± 2.03	101.89 ± 10.98	103.70 ± 4.60

Table S3. Mortality, morbidity and total number of clinical signs recorded per group of male and female rats for each dose level during the pharmacokinetic study. Four dose levels were tested: vehicle control (PK1), 2.5 (PK2), 5 (PK3) and 10 mg/kg (PK4).

Group	Sex	Total number of rats	Mortality (number of rats)	Morbidity (number of rats)	Total number of clinicals signs
PK1	Male	3	0	0	0
	Female	3	0	0	0
PK2	Male	9	0	0	0
	Female	9	0	0	0
PK3	Male	9	0	0	0
	Female	9	0	0	0
PK4	Male	9	0	0	0
	Female	9	0	0	0

Table S4. Concentrations of compound **5** detected in plasma for 24 h after an intravenous single dose injection in Wistar rats. Four dose levels were determined: vehicle control (PK1), 2.5 (PK2), 5 (PK3) and 10 mg/kg (PK4). ND = Non-Detectable.

Sex	Group	Mean Concentration (μg/L) at different timepoints (h)									
		0	0.08	0.25	0.5	1	2	4	6	10	24
Male	PK 1	ND	-	-	-	-	-	-	-	-	ND
	PK 2	ND	8,278.66 ± 267.62	3,906.51 ± 251. 86	1,609.63 ± 14,58	385.12 ± 76.33	82.98 ± 22.38	11.44 ± 0.43	4.67 ± 1.04	1.99 ± 0.84	4.02
	PK 3	ND	15,063.01 ± 1,865.49	8,033.35 ± 62.39	3,381.70 ± 163.03	753.62 ± 74.90	145.61 ± 40.57	22.97 ± 2.07	8.21 ± 1.85	1.95 ± 0.57	1.18
	PK 4	ND	26,316.93 ± 1,039.46	14,921.65 ± 812.42	6,712.58 ± 1,500.22	1,468.97 ± 220.75	361.74 ± 67.58	48.49 ± 10.22	17.30 ± 2.52	5.10 ± 0.86	1.49
Female	PK 1	ND	-	-	-	-	-	-	-	-	ND
	PK 2	ND	8,659.67 ± 398.25	3,914.53 ± 92.61	1,823.18 ± 217,74	472.01 ± 96.29	87.82 ± 9.76	14.59 ± 1.86	5.42 ± 1.14	1.58 ± 0.48	ND
	PK 3	ND	16,634.38 ± 2,671.52	7,175.47 ± 199.19	3,753.68 ± 791.95	941.97 ± 404.10	179.80 ± 7.72	23.10 ± 2.38	8.84 ± 3.45	2.69 ± 0.77	ND
	PK 4	ND	30,839.10 ± 1,014.03	15,048.98 ± 649.28	6,775.95 ± 1,715.28	1,957.85 ± 457.19	444.62 ± 90.61	62.65 ± 6.04	22.98 ± 3.24	9.08 ± 7.46	ND

Table S5. Pharmacokinetic parameters for an intravenous single dose injection of compound **5** in Wistar rats, male and female. Three dose levels were used: 2.5 (PK2), 5 (PK3) and 10 mg/kg (PK4). Parameters include maximum concentration (C_{max}), time of maximum observed concentration (T_{max}), area under the curve (AUC), clearance (Cl), volume of distribution (V_d), elimination constant (K_{el}), and elimination halftime ($t_{1/2}$).

Sex	Group	C_{max} (μg/L)	T_{max} (h)	AUC (μg·h/L)	Cl (L/h/kg)	V_d (L/kg)	K_{el} (h^{-1})	$t_{1/2}$ (h)
Male	PK 2	8,278.66	0.08	3,426.64	0.73	2.52	0.29	2.41
	PK 3	15,063.01	0.08	6,527.86	0.76	6.01	0.13	5.45
	PK 4	26,316.93	0.08	12,164.74	0.82	6.65	0.12	5.61
Female	PK 2	8,659.67	0.08	3,625.84	0.69	1.91	0.36	1.92
	PK 3	16,634.38	0.08	7,036.40	0.71	2.03	0.35	1.98
	PK 4	30,839.10	0.08	13,632.87	0.73	2.37	0.31	2.24

Table S6. Mortality, morbidity and clinical signs record of male and female Beagle dogs for the pharmacokinetic study during 24 h. A single dose level of 5 mg/kg b. wt. was tested.

Sex	Total number of dogs	Mortality (number of dogs)	Morbidity (number of dogs)	Clinical signs observed		
				N of dogs	Sign	Time period
Male	3	0	0	1	Mild vomiting	After dosing
Female	3	0	0	1	Mild vomiting	After dosing

Table S7. Pharmacokinetic parameters after an intravenous single dose injection of compound **5** in Beagle dogs, male and female. Dose level was 5 mg/kg b. wt.

Sex	C_{max} (µg/L)	T_{max} (h)	AUC (ng·h/L)	Cl (L/h/kg)	V_d (L/kg)	K_{el} (h^{-1})	$t_{1/2}$ (h)
Male	14,035.30	0.08	10,628.18	470.21	1,955.00	0.24	2.88
Female	15,771.13	0.08	11,549.84	432.62	1,894.77	0.23	3.04

Table S8. Mortality and morbidity record of male and female rats for each dose level group during the maximum tolerated dose (MTD) study for 14 days. Two dose levels groups: 300 mg/kg b. wt. (D1) and 500 mg/kg b. wt. (D2) were studied.

Group	Sex	Total number of rats	Mortality (number of rats)	Morbidity (number of rats)
D1	Male	5	0	0
	Female	5	0	0
D2	Male	5	0	0
	Female	5	0	0

Figure S1. Body weight of rats during the study period of MTD for each group and sex: 300 mg/kg b. wt. (D1) and 500 mg/kg b. wt. (D2). Each dose group per sex consisted of 5 rats.

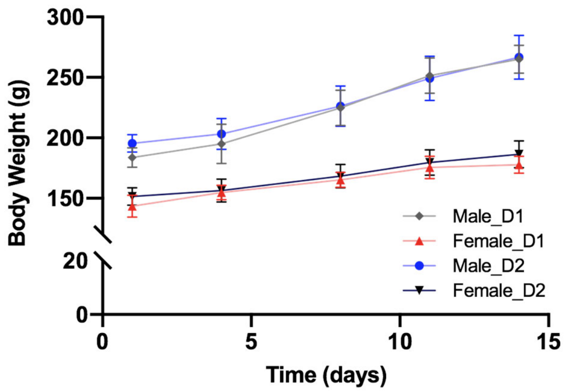

Table S9. Clinical signs observed in rats of dose level groups: 300 mg/kg b. wt. (D1) and 500 mg/kg b. wt. (D2) during an observation period of 14 days after injection. Each dose group consisted of 5 male and 5 female rats.

Clinical sign of toxicity	Sex	Number of rats showing signs	
		D1 (N=5)	D2 (N=5)
Erythema	Male	3	5
	Female	3	5
Necrosis	Male	0	2
	Female	0	5
Sloughing	Male	0	2
	Female	0	5

Table S10. Gross pathological findings observed in rats sacrificed at day 14 post dosing from groups 300 mg/kg b. wt. (D1) and 500 mg/kg b. wt. (D2). NAD = No Abnormality Detected.

Sex	Location	Organs/Lesions	Number of Gross Pathological Findings	
			D1 (N=5)	D2 (N=5)
Male	External	NAD	5	3
		Tail: Reddish discoloration	0	2
		Tail: Sloughing	0	1
	Internal	NAD	5	5
Female	External	NAD	5	0
		Tail: Reddish discoloration	0	5
		Tail: Sloughing	0	5
	Internal	NAD	5	5

Table S11. Mortality and morbidity record of male and female dogs for each group during the MTD study for 7 days. Two dose levels groups were studied: 50 mg/kg b. wt. (D3) and 75 mg/kg b. wt. (D4).

Group	Mortality			Morbidity		
	Male (N=1)	Female (N=1)	Total (N=1)	Male (N=1)	Female (N=1)	Total (N=1)
D3	0	0	0	0	0	0
D4	0	0	0	0	0	0

Table S12. Clinical signs presented for each individual dog during an observation period of 7 days after dosing: (**a**) clinical signs observed before dosing / morning; (**b**) clinical signs observed after dosing/ evening. Dose levels represent: 50 mg/kg b. wt. (D3) and 75 mg/kg b. wt. (D4). Each dose group consisted of 1 male and 1 female dogs.

a)

Dose level	Sex	Observation	Days observed		
			From day	To day	Total days
D3	Male	Normal	1	7	7
	Female	Normal	1	7	7
D4	Male	Normal	1	1	1
		Weakness	2	-	1
		Normal	3	7	5
	Female	Normal	1	1	1
		Weakness	2	-	1
		Normal	3	7	5

b)

Dose level	Sex	Observation	Days observed		
			From day	To day	Total days
D3	Male	Vomiting	1	-	1
		Normal	2	7	6
	Female	Vomiting	1	-	1
		Normal	2	7	6
D4	Male	Vomiting	1	-	1
		Weakness	1	2	2
		Normal	3	6	4
	Female	Vomiting	1	-	1
		Weakness	1	2	2
		Normal	3	6	4

Figure S2. Body weight evolution of dogs during the study period of MTD for each group and sex: 50 mg/kg b. wt. (D3) and 75 mg/kg b. wt. (D4). Each dose group per sex consisted of 1 dog.

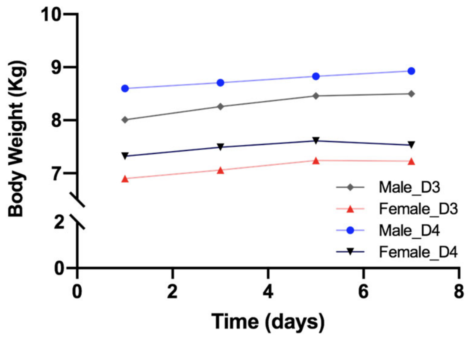

Table S13. Food consumption of D3 and D4 dogs during the study period of MTD. Each dose group per sex consisted of 1 dog. * = dogs were fasted for clinical pathology evaluation.

Group	Experimental day	Food consumption (g)	
		Male	Female
D3	1	300.1	300.3
	2	300.2	300.0
	3	300.0	300.1
	4	300.0	300.2
	5	300.2	300.1
	6	300.3	170.1*
	7	300.2	300.0
D4	1	300.1	300.3
	2	300.2	300.1
	3	300.5	300.3
	4	300.2	300.1
	5	300.0	300.2
	6	300.1	186.0*
	7	-	-

Table S14. Macroscopic and histopathological findings observed in 75 mg/kg b. wt. (D4) dogs sacrificed at day 7 post dosing. Organs not included in the table did not reveal any gross or histopathological findings. NAD = No Abnormality Detected.

**Sex**	**Macroscopic Findings**		**Microscopic Findings**
	**External**	**Internal**	
Male	NAD	NAD	**Liver:** mild cytoplasmic rarefaction, hepatocytes, centrilobular.<br>**Kidneys** (bilateral): minimal infiltration, mononuclear cells, interstitial, focal.<br>**Testes** (bilateral): immature – present.
Female	NAD	NAD	**Liver:** mild cytoplasmic rarefaction, hepatocytes, centrilobular.
